# Functional assessment of glioma pathogenesis by *in vivo* multi-parametric magnetic resonance imaging and *in vitro* analyses

**DOI:** 10.1038/srep26050

**Published:** 2016-05-20

**Authors:** Nai-Wei Yao, Chen Chang, Hsiu-Ting Lin, Chen-Tung Yen, Jeou-Yuan Chen

**Affiliations:** 1 Department of Life Science, National Taiwan University, Taipei, Taiwan; 2 Institute of Biomedical Sciences, Academic Sinica, Taipei, Taiwan; 3 Institute of Genome Sciences, National Yang-Ming University, Taipei, Taiwan

## Abstract

Gliomas are aggressive brain tumors with poor prognosis. In this study, we report a novel approach combining both *in vivo* multi-parametric MRI and *in vitro* cell culture assessments to evaluate the pathogenic development of gliomas. Osteopontin (OPN), a pleiotropic factor, has been implicated in the formation and progression of various human cancers, including gliomas, through its functions in regulating cell proliferation, survival, angiogenesis, and migration. Using rat C6 glioma model, the combined approach successfully monitors the acquisition and decrease of cancer hallmarks. We show that knockdown of the expression of *OPN* reduces C6 cell proliferation, survival, viability and clonogenicity *in vitro*, and reduces tumor burden and prolongs animal survival in syngeneic rats. *OPN* depletion is associated with reduced tumor growth, decreased angiogenesis, and an increase of tumor-associated metabolites, as revealed by T2-weighted images, diffusion-weighted images, K^trans^ maps, and 1H-MRS, respectively. These strategies allow us to define an important role of OPN in conferring cancer hallmarks, which can be further applied to assess the functional roles of other candidate genes in glioma. In particular, the non-invasive multi-parametric MRI measurement of cancer hallmarks related to proliferation, angiogenesis and altered metabolism may serve as a useful tool for diagnosis and for patient management.

Gliomas are the most common primary tumors of the brain. Despite recent advances in surgery and clinical neuro-oncology, the prognosis of patients with gliomas remains poor^1^. Gliomas, similar to other human cancers, are resulted from the accumulation of genetic and epigenetic alterations that drive the conversion of normal cells to malignant cells. Similarly, the multi-step pathogenic development of glioma is accompanied with the acquisition of characteristic phenotypes of malignant traits described as cancer hallmarks^2^,^3^. Six biological capabilities were initially proposed as the manifestation of the vast complexity of the changes of all types of tumors, including self-sufficiency in growth signals, insensitivity to anti-growth signals, evading apoptosis, limitless replicative potential, sustained angiogenesis and tissue invasion and metastasis^2^. Later on, additional traits such as genomic instability, evasion of immune surveillance, inflammation, and altered metabolism have also emerged^3^. A strategy to monitor the development of these common traits will help to identify the candidate genes involved in glioma pathogenesis, and further to provide useful markers for diagnosis, prognosis and patient management. Magnetic resonance imaging (MRI) provides a non-invasive *in vivo* assessment of the progression of malignant transformation in a timely and efficient manner^4^. By using multi-parametric MRI methods, patho-physiologic parameters regarding tumor location, tumor volume, and the degree of angiogenesis can be easily monitored in glioma pathogenesis. In addition, proton magnetic resonance spectroscopy (1H-MRS) allows quantitative measurement of the changes in tumor-associated metabolites at the lesion site. We proposed in this study that *in vivo* MRI assessment plus *in vitro* characterization of the functions of candidate genes in promoting cancer hallmarks may constitute a powerful and straightforward strategy to assess the roles of key molecular players involved in the pathogenic development of gliomas.

Osteopontin (OPN), also named secreted phosphoprotein 1 (SPP1), is a member of the small integrin-binding ligand N-linked glycoprotein (SIBLING) family^5^. OPN was initially identified as an inducible marker for epithelial transformation^6^. OPN expression is frequently elevated in human cancers and elevated expression of OPN is associated with poor prognosis^7^. In human glioma, OPN expression was correlated with tumor grade and serum levels of OPN were correlated with poor prognosis^8^. In animal models, *OPN* was identified as one of the highly expressed genes in rat C6 gliomas^9^ and in N-ethyl-nitrosourea (ENU)-induced gliomas^10^. OPN expression was demonstrated to affect cell proliferation, survival, invasion, and angiogenesis^7^. KD of *OPN* led to anti-tumorigenic effect in several types of cancers, including breast cancer^11^ and lung cancer^12^. However, the roles of OPN in gliomas have not been fully addressed. Lamour *et al*. have shown that KD of *OPN* expression in human U87 cells suppressed xenografted tumor formation in the chicken chorio-allantoic membrane model^13^. Similarly, Jan *et al*. also demonstrated that KD of *OPN* in U87 cells greatly reduced the growth of xenografted tumors in nude mice^14^. In this study, we adopted an allografted rat C6 glioma model to assess the function of OPN in the process of glioma pathogenesis. Rat C6 glioma cells proficient and deficient in OPN expression were *in vitro* characterized and also intracranially implanted into the rat brains followed by MRI assessment of tumor formation and tumor-associated malignancies.

As a proof-of-concept study, here, we addressed the role of OPN in glioma pathogenesis using *in vitro* and *in vivo* tumorigenic assays. We demonstrated a close link of OPN to the acquisition of several cancer hallmarks in gliomas. OPN expression was associated with tumor growth, angiogenesis, and altered metabolism. The combined strategy thus provides new concept for evaluation of cancer hallmarks in gliomas in both clinical and pre-clinical studies.

## Results

### KD of *OPN* expression reduces C6 glioma cell viability, proliferation, survival, and clonogenicity

Using OPN as a model molecule, this study aimed to establish a straightforward approach to assess the involvement of candidate genes in promoting the pathogenic development of gliomas by monitoring the acquisition of various cancer hallmarks. Rat C6 glioma cell line, a well-established and -documented glioma model^15^,^16^, was chosen as a platform for establishing the *in vitro* and *in vivo* assessments to monitor tumor growth, tumor invasion, angiogenesis and metabolic changes.

**Figure 1:**
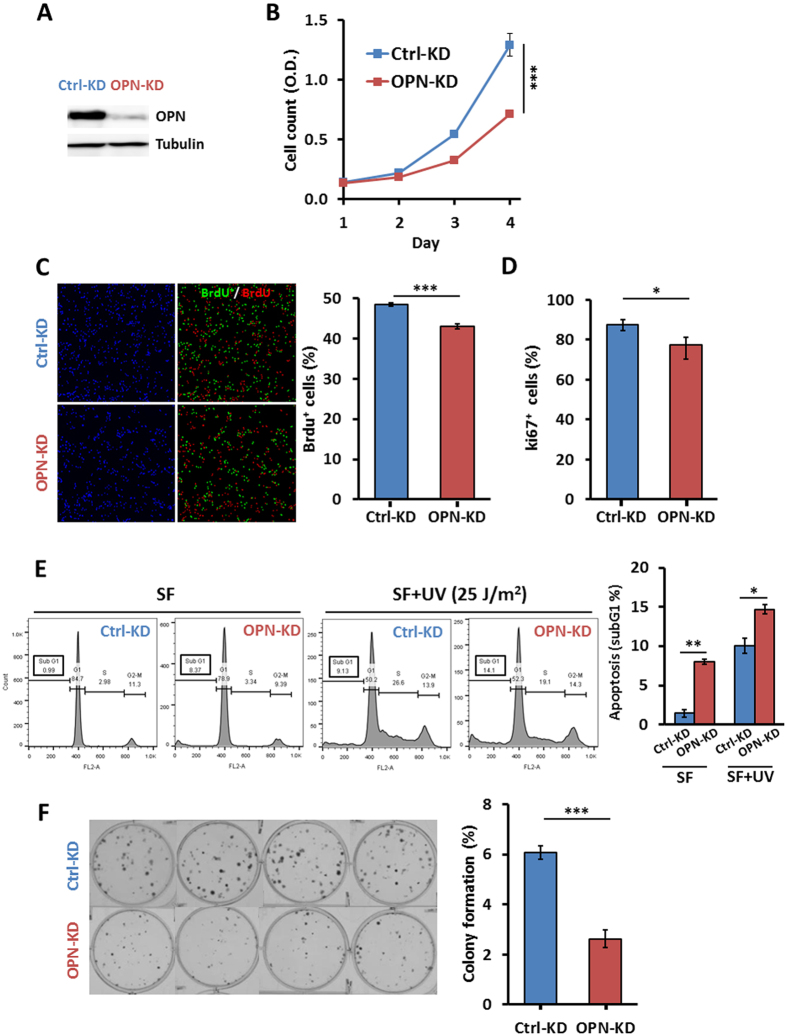
KD of OPN expression reduces cell viability, proliferation, survival, and clonogenicity in C6 glioma cells. (**A**) The expression of OPN in rat C6/Ctrl-KD and C6/OPN-KD glioma cells was examined by western blotting with tubulin as a loading control. (**B**) The C6/Ctrl-KD and C6/OPN-KD cells were subjected to cell viability assay using Cell Counting Kit-8 assay. (**C**) Cells were subjected to BrdU incorporation for 45 min followed by staining for BrdU. (**D**) Cells were subjected to Ki-67 staining. (**E**) Cells were subjected to apoptosis assay. Cells were treated with or without UV irradiation at 25 J/m^2^ and incubated in serum-free medium for 24 hrs, and harvested for FACS analyses of sub-G1 fractions. (**F**) Cells were subjected to colony formation assay. Data are derived from three experiments and presented as means ± SEM. *p < 0.05; **p < 0.01; ***p < 0.001 by ANOVA analysis (Cell Counting Kit-8 assay) and student’s two-tailed *t*-test (proliferation assay, colony formation assay and UV-induced apoptosis assay).

To examine the role of OPN in the pathogenic development of gliomas, loss-of-function approach was taken to examine the effects of *OPN* depletion on the acquisition of cancer hallmarks. C6/OPN-KD cell clone, which stably harbored shRNA against *OPN*, and C6/Ctrl-KD cell clone harboring the control shRNA, were established, respectively. The efficiency of OPN KD was demonstrated by western blot analysis (Fig. 1A). By Cell Counting Kit-8 assay, we showed that KD of *OPN* led to a significant decrease in C6 cell viability (Fig. 1B, ANOVA: F(1,40) = 37.5, *p* < 0.0001; Fisher’s post-hoc tests: *p* < 0.0001). The decreased cell viability was due to reduced cell proliferation and increased apoptosis. As shown, KD of *OPN* caused a decrease of cells in the S phase as demonstrated by BrdU incorporation assay, the marker of DNA synthesis (Fig. 1C, Student *t* tests: *p* = 2.32 × 10^−4^). Similarly, a decrease of cycling cells was observed in *OPN*-depleted cells by Ki-67 staining, the marker of cellular proliferation (Fig. 1D; Student *t* tests: *p* = 4.91 × 10^−2^). In addition, KD of *OPN* led to increased sensitivity toward apoptosis induced by serum deprivation alone or together with UV irradiation (Fig. 1E), suggesting an anti-apoptotic role of OPN in glioma cells. Most importantly, KD of *OPN* reduced the ability of C6 cells in forming colonies (Fig. 1F; Student *t* tests: *p* = 2.05 × 10^−5^).

### KD of *OPN* expression reduces tumor growth and prolongs survival in rat glioma model

**Figure 2:**
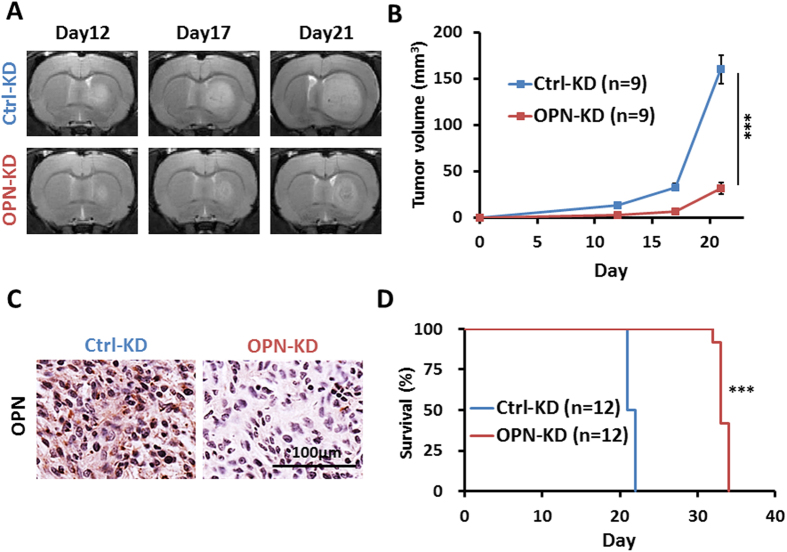
KD of OPN expression reduces *in vivo* tumor growth and prolongs survival. The rat C6/OPN-KD and C6/Ctrl-KD cells were implanted into rat brains by intracranial injection, and tumor growth was monitored by T2WI at 12, 17, and 21 days after implantation. (**A**) Representative images of C6/OPN-KD and the C6/Ctrl-KD cell-derived tumors were shown. (**B**) The outline of the tumor was delineated based on the contrast between the brain tissue and the tumor area. Volumes of the tumors at different time points were estimated using T2WIs (n = 3 for each group in each experiment, and 3 independent experiments were performed). (**C**) Brain sections of the animals sacrificed immediately after the scans on day 21 were subjected to immunohistochemical staining of OPN. (**D**) The survival of the tumor-bearing rats was followed after implantation (n = 6 for each group in each experiment and 2 independent experiments were performed). Data are presented as means ± SEM. *p < 0.05; **p < 0.01; ***p < 0.001 by student’s two-tailed *t*-test.

We further investigated the role of OPN in tumor development *in vivo* using the intracranial rat C6 glioma model. The OPN-KD and the Ctrl-KD C6 cells were implanted into rat striatum, and tumor growth was followed by longitudinal T2WI scans. As shown in Fig. 2A,B, KD of *OPN* greatly reduced the tumor formation of grafted C6 cells as followed by T2WI measurement on days 12, 17 and 21 after implantation (ANOVA: F(1,48) = 98.1, *p* < 0.0001; Fisher’s post-hoc tests: *p* < 0.0001). Immunohistochemical analysis of OPN expression in animals sacrificed on day 21 confirmed the significantly reduced expression of OPN in the brain tumors derived from C6/OPN-KD cells (Fig. 2C). In accordance to the delayed tumor progression, animals bearing OPN-deficient tumors displayed significantly prolonged survival (Fig. 2D; Student *t* tests: *p* = 3.65 × 10^−23^). These results suggest that OPN plays an important role in the pathogenic development of C6 glioma.

### OPN deficiency is associated with decreased glioma malignancies

**Figure 3:**
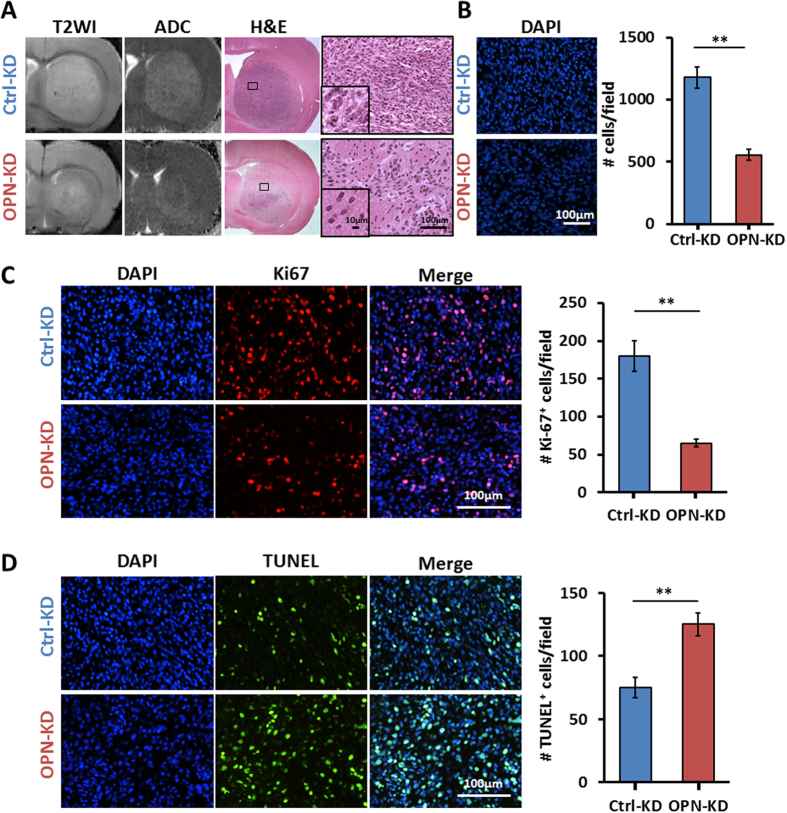
OPN deficiency leads to decreased tumor cellularity, accompanied with decreased proliferation and increased apoptosis, in gliomas. The rat C6/OPN-KD and C6/Ctrl-KD cells were implanted intracranially into 9-week-old Sprague-Dawley rats. The animals were sacrificed on day 21 and brain sections were subjected to T2WI image analysis and immunohistochemical examination. (**A**) Representative images of T2WI, ADC maps, and H&E staining of C6/OPN-KD- and C6/Ctrl-KD-derived tumors are shown. The nuclear morphology was shown in magnified images in the right panels and in the insets. (**B**) Tumor cellularity was evaluated by nuclear staining by DAPI. (**C**) Tumor cell proliferation was examined by staining of Ki-67 (red) followed by counterstaining with DAPI (blue). (**D**) Tumor cell apoptosis was examined by TUNEL staining (green) followed by counterstaining with DAPI (blue). All the representative images are shown at 40× magnification. Quantification was performed by counting positive cells in four images captured from each animal of four animals examined per group. Data were derived from 3 independent experiments, and are presented as means ± SEM in the bar graph. *p < 0.05; **p < 0.01; ***p < 0.001 by student’s two-tailed *t*-test.

Tumor cellularity is an important feature in histologic grading of gliomas^17^. High-grade gliomas are characterized by prominent hypercellularity, increased mitotic activity, and nuclear atypia (nuclear enlargement, pleomorphism and hyperchromasia). In consistence to previous data, KD of OPN yielded tumors with smaller size as shown by T2WI (Fig. 3A, left panel). H&E staining revealed that the reduced tumor growth from the OPN-deficient C6 xenograft was associated with a dispersed, loose arrangement of the tumor cells, in contrast to the compact arrangement of the cells in the OPN-proficient tumors (Fig. 3A, right panels). In addition, the OPN-proficient tumor cells were characterized with enlarged, irregular shaped, variably hyperchromatic nuclei, whereas the OPN-deficient tumor cells displayed smaller nuclei with less nuclear atypia (Fig. 3A, insets). These data suggest that KD of *OPN* in C6 cells led to tumor formation with less aggressive neoplastic nuclear features. DAPI staining allowed the quantitation of the cell density, which clearly demonstrated the reduced cellularity in the OPN-deficient tumors (Fig. 3B; Student *t* tests: *p* = 1.03 × 10^−3^), further confirmed that depletion of *OPN* significantly reduced tumor malignancy. Apparent diffusion coefficient (ADC) measures the diffusion of water, and the increment of ADC value is frequently inversely correlated with the increment of tumor cellularity in areas of solid tumor with some exceptions^18^. In this study, we show that the OPN-proficient tumors displayed increased ADC when tumor cell density was increased (Fig. 3A, middle left panel), suggesting the possibility of factors facilitating the local diffusion in the OPN-proficient tumor tissues.

We further examined whether the reduced tumor burden and tumor-malignancy observed in the OPN-deficient tumors was due to the effects of OPN depletion on cell proliferation and apoptosis. Ki-67 and TUNEL staining were performed and the representative images were shown in Fig. 3C,D, respectively. Few positive signals of Ki-67 and TUNEL staining were detected in the normal brain sections (data not shown), but the Ki-67 and TUNEL signals were readily detected in the tumors derived from the C6/Ctrl-KD cells. In the OPN-deficient tumors, the number of Ki-67^+^ cells was significantly reduced (Student *t* tests: *p* = 8.39 × 10^−3^ of Ki-67 staining). On the other hand, a significant increase in the number of TUNEL^+^ cells was observed in the OPN-deficient tumors (*p* = 6.25 × 10^−3^ of TUNEL staining). These *in vivo* data further confirmed the *in vitro* observations that OPN depletion is associated with reduced cell proliferation and increased apoptosis.

### KD of *OPN* suppresses tumor angiogenesis

**Figure 4:**
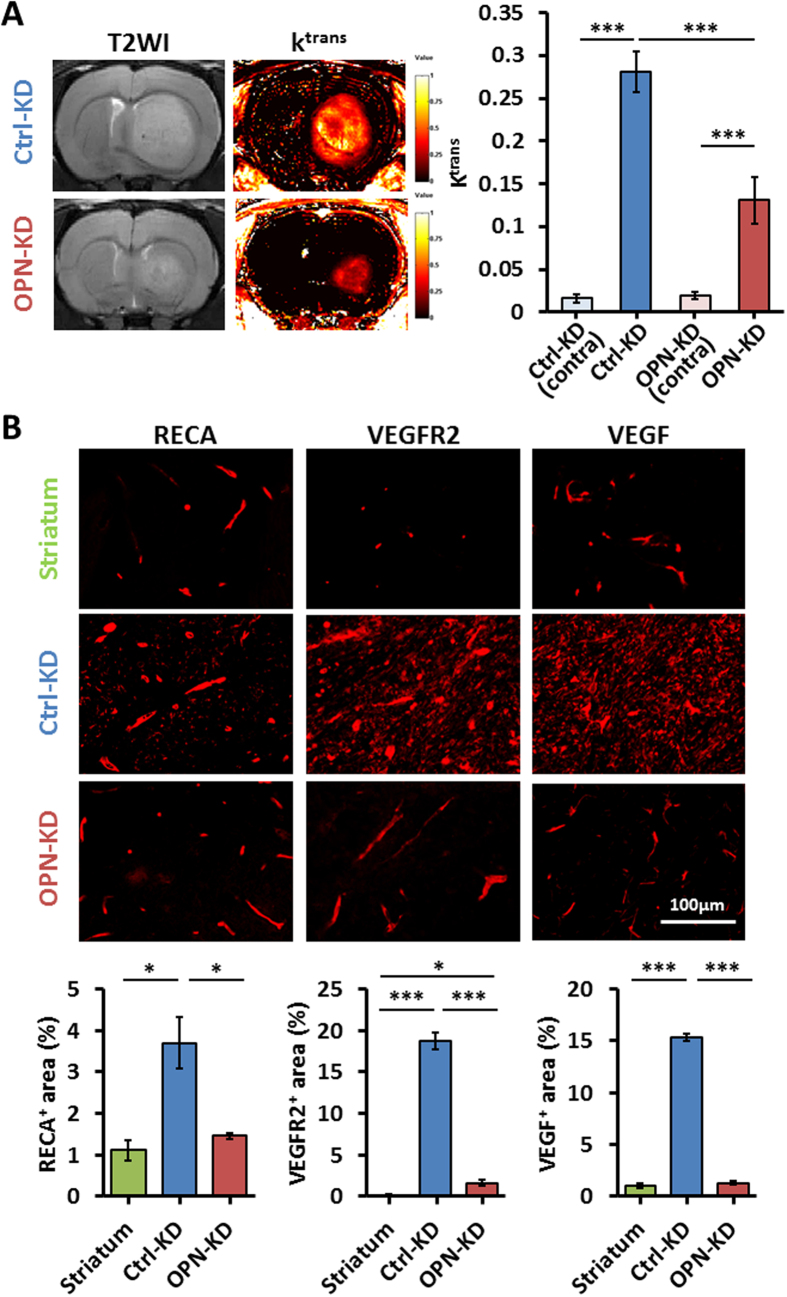
KD of OPN expression is associated with significantly decreased angiogenesis in glioma. Rat C6/OPN-KD and C6/Ctrl-KD cells were implanted into the rat brains, and tumor phenotypes were monitored *in vivo* by T2WIs and K^trans^ maps on day 21. (**A**) Representative MR images of C6/OPN-KD- and C6/Ctrl-KD-derived tumors are shown. The K^trans^ values in tumor regions, which were delineated based on the contrast provided by the T2WIs between the tumor and the brain tissues, were quantified and plotted. The color ranged from black (0/min), orange (0.5/min), to white (1/min). (**B**) Tumor vessel density was evaluated by staining RECA. Expression of angiogenic markers was examined by staining VEGFR2 and VEGF. All the representative images are shown at 40× magnification. Quantification was performed by calculating the coverage of positive signals in four images captured from each animal of four animals examined per group. Data are presented as means ± SEM. *p < 0.05; **p < 0.01; ***p < 0.001 by student’s two-tailed *t*-test.

Hyper-neovascularization is a hallmark associated with advanced gliomas and with adverse prognosis^19^. The newly formed blood vessels in the brain tumors are structurally and functionally abnormal. These vessels have irregular diameters, being fragile, leaky, and with compromised blood–brain barrier (BBB) function. By monitoring the perfusion and permeability of vessels in tumor vascular bed, the vascular transfer constant (K^trans^) maps, generated from dynamic contrast-enhanced MRI (DCE-MRI), serves as a vital tool to investigate the degree of angiogenesis^20^. Figure 4A shows the T2WI and K^trans^ maps of the tumors on day 21 after implantation of C6/Ctrl-KD and C6/OPN-KD cells (Fig. 4A). The K^trans^ signals of the contralateral normal striatum in the C6/Ctrl-KD and OPN-KD animals were low. No significant difference was found in the K^trans^ maps derived from the contralateral striatum in both groups (ANOVA: F(1,16) = 0.21, *p* > 0.05; Fisher’s post-hoc tests: *p* = 0.6528). Tumor formation was high-lighted with significantly increased K^trans^ signals in both the OPN-proficient and -deficient tumors (all *p* values <0.0001 for tumor and its contralateral site). Importantly, KD of OPN greatly reduced the K^trans^ signals in the OPN-deficient tumors as compared to the OPN-proficient tumors (Fig. 4A, bar graph; Student *t* tests: *p* = 3.43 × 10^−6^). These data suggest that *OPN* depletion delayed tumor growth in part through reducing angiogenesis. In support, immunohistochemistry of endothelial marker RECA revealed widespread vessel formation in the tumors developed from C6/Ctrl-KD cells as compared to the normal brain (Fig. 4B, left column, top and middle panels), and that KD of *OPN* significantly suppressed vessel formation in the tumors (Fig. 4B, left column, middle and bottom panels). In consistence to the lowered density of vessels observed in the OPN-deficient tumors, significant decreased expression of angiogenic markers, particularly VEGFR2 and VEGF, were also observed in the OPN-deficient tumors and the tumor microenvironment (Fig. 4B, middle and right columns, Student *t* tests: *p* = 1.51 × 10^−4^ for VEGFR2, *p* = 1.42 × 10^−6^ for VEGF). These results suggest that KD of *OPN* adversely impacts on tumor angiogenesis.

### KD of *OPN* expression is associated with changes in tumor-associated metabolites

**Figure 5:**
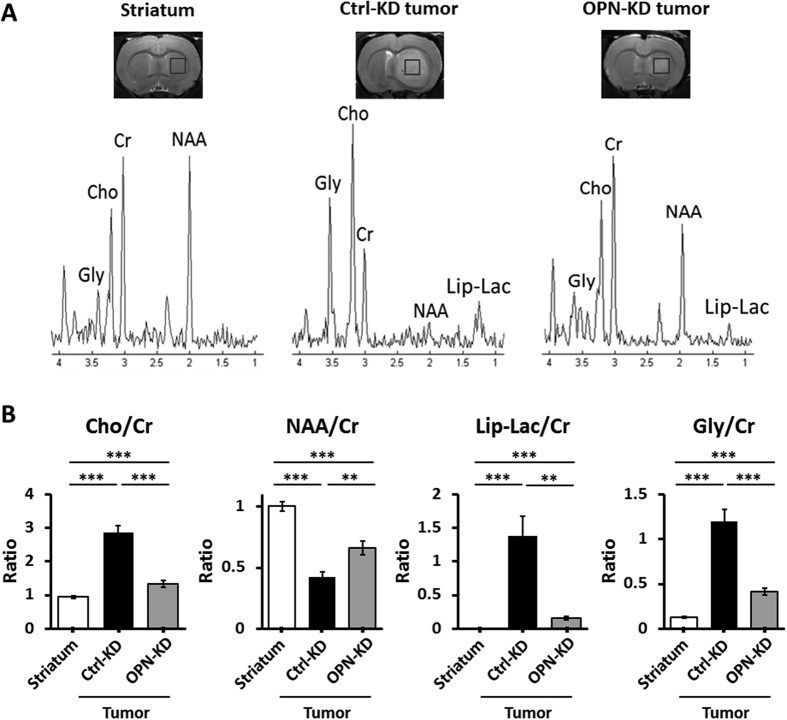
KD of OPN expression is associated with changes in tumor-associated metabolites. (**A**) Axial T2WI showing the location of the voxel selected for volume localized spectroscopy. The voxel was localized at the center of tumor mass. Representative proton MR spectra identifying the signals corresponding to N-acetylaspartate (NAA), total creatine (Cr), lipid and lactate (Lip-Lac), choline (Cho), and glycine (Gly) were accessed in both C6/OPN-KD-derived and C6/Ctrl-KD-derived tumors described in Fig. 2. The presence of NAA may be associated with decreased de-differentiation process in the OPN-deficient tumors or be in part due to the partial volume effect that results in contributions from normal brain tissues. Striatum from normal rat was included as control. Three animals were examined for each group in each experiment and three independent experiments were performed. (**B**) The integral ratios of Lip-Lac/Cr, NAA/Cr, Cho/Cr, and Gly/Cr were analyzed by using LC model software. Data are presented as means ± SEM. *p < 0.05; **p < 0.01; ***p < 0.001 by student’s two-tailed *t*-test.

Single-voxel 1H-MRS was performed on day 21^st^. In comparison to the MR spectra of the normal brain, the tumor spectra have been reported to exhibit a higher Cho/Cr ratio due to increased proliferation and a lowered NAA/Cr ratio due to loss of neurons^21^ or neural dedifferentiation^22^. In addition, the combined signals of mobile lipid signal and lactate (Lip-Lac) has been demonstrated associated with occurrence of necrosis/glycolytic switch in the cancer, which were both vital markers of malignancy during tumor development^22^,^23^. Increased glycine concentration in the brain has been shown to be associated with altered metabolism in the cancer and was proposed as a marker for grade IV gliomas^24^,^25^. As shown in Fig. 5A, distinct patterns of MR spectra were found in the normal brain and tumors derived from C6/Ctrl-KD and C6/OPN-KD cells. In comparison to the normal striatum, tumors derived from the C6/Ctrl-KD cells displayed significantly increased Cho/Cr and decreased NAA/Cr ratios as expected (Fig. 5B; Student t tests: *p* = 3 × 10^−5^ for Cho/Cr and *p* = 2.35 × 10^−3^ for NAA/Cr). Furthermore, elevated production of lipid-lactate and glycine was also demonstrated in the tumors (Fig. 5B; Student *t* tests: *p* = 8.95 × 10^−4^ for Lip-Lac/Cr and *p* = 2.1 × 10^−5^ for Gly/Cr). Most importantly, KD of *OPN* reversed the changes in these tumor-associated metabolites. In comparison to the OPN-proficient tumors, OPN-deficient tumors displayed decreased ratios of Lip-Lac/Cr and Gly/Cr (Fig. 5B; Student *t* tests: *p* = 1.82 × 10^−3^ for Lip-Lac/Cr and *p* = 1.5 × 10^−4^ for Gly/Cr), along with decreased Cho/Cr and increased NAA/Cr ratios (Fig. 5B; Student t tests: *p* = 3 × 10^−5^ for Cho/Cr and *p* = 2.35 × 10^−3^ for NAA/Cr). The higher levels of NAA observed in the OPN-KD tumors were associated with higher numbers of cells stained positive for the neuronal markers NeuN and β-tubulin, suggesting a decreased de-differentiation process in the OPN-KD tumors, as compared to the Ctrl-KD tumors (Supplementary Fig. S1). However, we cannot rule out the possibility of a partial volume effect that the NAA spectra may be in part due to the presence of normal brain tissues surrounding the tumor tissue. No significant difference was observed in the MRS profiles of the contralateral normal striatum of the animals bearing OPN-KD and Ctrl-KD tumors (data not shown). These results suggest that KD of *OPN* tuned down the process of malignant transformation and decreased tumor malignancy.

In summary, we have shown in the study that a combined approach of *in vivo* multi-parametric magnetic resonance imaging and *in vitro* analyses demonstrated that OPN plays an important role in promoting the pathogenesis of glioma. KD of OPN in C6 cells significantly reduced the rate of xenograft tumor growth, yielding a prolonged survival. In support, we showed that OPN KD-mediated retardation of tumor development was associated with reduced cell proliferation, increased programmed cell death, and reduced angiogenesis. We further confirmed that KD of OPN retards tumor growth by examining the images and histology-related parameters in C6/Ctrl-KD cell- and C6/OPN-KD cell-derived tumors of similar size at both early and late stages. As shown in the Supplementary Fig. S2, OPN depletion resulted in reduced tumor cell proliferation, reduced cellularity, nuclei atypia, reduced angiogenesis at the early stage of tumor development. After extended time, the OPN-KD tumor, although grew much slower than the Ctrl-KD tumor, eventually gave rise to large tumor while reaching late stage.

## Discussion

Using the rat C6 glioma cell line, a well-established and -documented glioma model^15^,^16^, in this study, we established a novel strategy combining *in vitro* and *in vivo* assessments to define the role of OPN in glioma pathogenesis. The combined strategy allows straightforward assessments of cancer hallmarks of proliferation, survival, angiogenesis and altered metabolism, and can be further applied to address the involvement of other candidate genes in glioma pathogenesis. Using *in vitro* characterization, we showed that depletion of *OPN* reduces tumor cell proliferation, survival, viability and clonogenicity. Using multi-parametric MRI methods to monitor *in vivo* tumor development in the rat C6 glioma model, we demonstrated that KD of *OPN* led to reduced tumor growth, decreased angiogenesis, and an increase of tumor-associated metabolites, which were closely associated with prolonged survival. Similar findings were also found using human U87-MG cells as a platform for testing the effect of OPN KD in the studies of others^14^ and our unpublished data. In conclusion, our findings support the notion that OPN plays an important role in glioma pathogenesis, and that OPN may serve as a marker for glioma therapy and management.

OPN, a member of the SIBLING family proteins, is constitutively secreted in a variety of tissues and upregulated in the sites of inflammation and malignant lesions^26^. OPN was first discovered in the bone as an extracellular matrix protein, which regulates osteoblast function during bone formation. In addition to bone remodeling, OPN is involved in multiple cellular functions including apoptosis, cellular proliferation, angiogenesis, cell migration, and adhesion^7^. OPN exerts its effects through interacting with cell surface receptors CD44 and integrins^7^. OPN was also identified as a protein secreted by malignant epithelial cells^6^, and its expression was further demonstrated to be closely associated to tumor progression in many human cancers. Recently, several studies have implicated OPN as a key molecule in glioma pathogenesis^13^,^14^.

In this study, 1H-MRS was acquired with a 136 ms echo time (TE). In such spectra, mobile lipid signal (1.26 ppm) overlaps the lactate resonance (1.3 ppm). The spectra at approximately 1.3 ppm were composed of lipid and lactate signals. The lipids are mainly due to occurrence of necrosis during tumor progression^23^ and lactate is mainly due to anaerobe metabolism. During malignant transformation, energy metabolism is switched from an oxidative to glycolytic bioenergetics pathway, even under aerobic condition, which has been described as Warburg effect^27^. The altered metabolism, a trait shared by virtually all cancer cells, leads to the production of lactate. High-grade gliomas were shown to have increased lactate content in comparison with low-grade gliomas^22^, thus, MRS has been suggested to be useful in differentiating the degree of malignancy in gliomas by evaluating the lactate content^28^. In the present study, an apparent increase in the lactate content was demonstrated during tumor formation from the implanted C6 cells. Most importantly, a significant decrease of lactate was found in tumors deficient in OPN, suggesting that KD of *OPN* greatly suppressed tumor malignancy. It has been proposed that the lower efficiency of energy production in glycolytic pathway is accompanied with increased production of small molecules, which serve as building blocks for protein, fatty acid, and nucleotide synthesis required for tumor growth. Glycine (Gly), the simplest non-essential amino acid, is one of small molecules with vital functions in the mammalian brain, mainly serving as a neurotransmitter and neuromodulator. Gly can be synthesized through several pathways, and is predominantly derived from its precursor serine from 3-phosphoglycerate through glycolysis. Several enzymes involved in Gly synthesis through glycolysis have been shown to be associated with increased proliferation in various types of cancer cell lines^29^ including glioma^30^. The expression of PHGDH was correlated to the aggressiveness of gliomas^30^. 1H-MRS studies have demonstrated the usefulness of glycine as a biomarker of malignancy in childhood brain tumors^31^, and for discriminating high-grade from low-grade gliomas. In this study, we demonstrated that the non-invasive 1H-MRS allowed the evaluation of the levels of glycine in gliomas, and that a dramatic increase of Gly was observed upon C6 glioma formation, concomitant to lactate production. Furthermore, OPN depletion led to a significant decrease of Gly, along with the decreased production of lactate. Other than quantitative evaluation of the production of lactate and glycine in gliomas, the alterations of several tumor-associated metabolites, such as choline and NAA, were also readily detected by 1H-MRS. Elevated choline has been shown to be tightly linked to an increase in cell membrane turnover during cell proliferation, and an increased Cho/Cr ratio is considered to be associated with malignant growth^32^. On the other hand, decreased levels of NAA may reflect the dedifferentiation of the neurons during malignant transformation^22^, and a decreased NAA/Cr ratio is consistent with the replacement of healthy neurons to neoplastic cells during malignant transformation^21^. Several reports have demonstrated the usefulness of the relative levels of metabolites, such as Cho/Cr^33^ and NAA/Cr^34^, in distinguishing low-grade from high-grade gliomas. In our study, a significant increase in Cho/Cr and a decrease in NAA/Cr ratios were associated with highly proliferative status of tumor formation, and KD of *OPN* in C6 cells greatly reversed the changes of these metabolites.

In addition to metabolic and proliferative hallmarks, we also showed that expression of OPN greatly influenced the angiogenic hallmark of gliomas. C6 glioma cells implanted into rat brains have been shown to form vascularized tumors in a VEGF-dependent manner^35^. In our study, we demonstrated the significant angiogenesis during the growth of C6 glioma by K^trans^ maps, which was accompanied with concomitant up-regulation of several angiogenic factors, in particular VEGF and VEGFR-2. We further showed that KD of *OPN* significantly decreased vascular perfusion and permeability in the C6-derived tumors, suggesting a role of OPN in regulating tumor vascularization, in part mediated through the regulation of VEGF and VEGFR2 expression.

Diffusion MRI is sensitive to tissue microarchitectural alterations and has become an attractive means for characterization of tumor cellularity. It is generally considered that the ADC value is inversely correlated with the degree of tumor cellularity^36^, since increased cellularity reduces the extracellular compartment, thus impeding the free translational movement of water molecules. However, due to the fact that ADC values derived from the conventional monoexponential model may be affected by a number of biological parameters in the tumor microenvironment, especially the perfusion component, some exceptions have been reported^18^,^36^,^37^,^38^,^39^,^40^. This is probably because the ADC values derived from the conventional monoexponential model may be affected by a number of biological parameters in tumor microenvironment, especially the perfusion component. Muti *et al*. reported that the value of the ADC was elevated in the solid part of tumor in subset of glioblastoma patients despite of the increased cellularity^18^, and that the increased ADC signal was correlated with the increment of edema (% of T1-signal Gd-enhancement) in the areas of solid tumor without necrosis. It was suggested that the presence of edema in solid tumor regions may cause an increase in the extracellular compartment and compensate the effect of the increase of cellularity^41^,^42^. In this study, we showed that OPN-proficient tumors displayed significantly increased ADC values than the OPN-deficient tumors (n = 9; *p* < 0.001), despite of its high cellularity. These data suggest the presence of other factors or cellular context that may facilitate local diffusion. In support, we showed that OPN-proficient tumors exhibited significantly increased K^trans^ maps (n = 9; *p* < 0.001), suggesting that the increased diffusion and ADC values may be facilitated in part due to the leakage of vessels in the tumor microenvironment. The recently established diffusion MRI models, such as intravoxel incoherent motion (IVIM)^41^, could help to resolve this issue. The biexponential IVIM model can estimate the effects of diffusion as well as perfusion, respectively, and provide a more accurate quantitative analysis of the cellularity and perfusion of tumors^43^,^44^,^45^,^46^,^47^. The IVIM model has therefore been demonstrated to be useful in predicting glioma grading^43^,^44^,^45^,^46^. All these studies warrant further investigation of the functional implications and differential assessment of ADC signals in various cellular contexts in glioblastomas using various diffusion models.

In conclusion, our findings suggest that OPN is a major molecular player associated with the pathogenic development of gliomas. The *in vitro* and *in vivo* assessments allow us to demonstrate the roles of OPN in conferring cancer hallmarks related to proliferation, survival, angiogenesis, and altered energy metabolism. This approach can be further applied to address the involvement of other candidate genes in glioma pathogenesis.

## Materials and Methods

### Antibodies and cell line

Anti-OPN antibodies for western blotting and immunohistochemical staining were purchased from Santa Cruz Biotechnology (Santa Cruz, CA, USA) and Developmental Studies Hybridoma Bank (DSHB, IA, USA), respectively. Anti-Ki-67 and anti-VEGFR2 were from Cell Signaling Technology (MA, USA). Rabbit anti-vascular endothelial growth factor (VEGF) antibody was from Thermo Scientific (MA, USA). Mouse anti-rat endothelial cell antigen (RECA) antibody was from Abcam (MA, USA).

Rat C6 glioma cell line was purchased from the Bioresource Collection and Research Center (ATCC ^#^CCL-107, BCRC, Taipei, Taiwan). The cells were cultured in Dulbecco’s modified Eagle’s medium (DMEM; Gibco, NY, USA) supplemented with 10% FBS (Gibco) and 1% penicillin-streptomycin (Gibco), and maintained under tissue culture condition at 37 °C with 95% air, 5% CO_2_, and 100% humidity.

### Lentivirus-based shRNA KD approach

The pLKO.1-shRNA plasmids encoding shRNAs targeting rat *OPN* (TRCN0000009605: 5′-ACTCGGATGAATCTGACGAAT-3′) and firefly luciferase (TRCN0000072246: 5′-CAAATCACAGAATCGTCGTAT-3′) were purchased from the National RNAi Core Facility, Taipei, Taiwan. Briefly, pLKO.1-shRNA plasmid was co-transfected with packaging plasmids pMD.G and pCMVΔR8.91 into 293T cells by LF2000 (Gibco). After 48 hours, the media containing lentivirus were harvested. To establish C6/Ctrl-KD and C6/OPN-KD cell clones, C6 cells were infected with the collected viruses (MOI = 2) in the presence of polybrene (8 ml) for 14 days as described previously^48^.

### Western blot analysis

Cells were lysed in RIPA buffer (10 mM Tris-HCl, pH 7.4, 150 mM NaCl, 1% Triton X-100, 1% sodium deoxycholate, 0.1% SDS, 1 mM EGTA, 1× protease cocktail, 1 mM NaF, 1 mM Na_3_VO_4_, and 1 mM DTT). The protein concentration was determined by Bradford protein assay (Bio-Rad, CA, USA). Proteins were separated by SDS-PAGE, transferred to PVDF membranes, and probed with the indicated antibodies. The immunoreactive bands were detected by chemiluminescent assay using Immobilon Western Chemiluminescent HRP substrate (Millipore, MA, USA).

### Cell viability, proliferation, apoptosis and colony formation assays

Cell viability assays were performed by seeding 1 × 10^3^ cells in each well of 96-well plates, with fresh medium replenished every other day. Cell number was measured at designated times using Cell Counting Kit-8 reagent (Sigma-Aldrich, MO, USA).

Cell proliferation assays were performed by seeding 5 × 10^4^ cells in each well of 24-well plates. After 24 hours, cells were incubated with 10□M BrdU for 45 min, and BrdU incorporation was detected by immunofluorescence using mouse anti-BrdU (Clone Bu20a, Dako, Denmark) and Alexa Fluor 488 conjugated secondary antibody, and subjected to the ImageXperss Micro XL high Content Imaging System (Molecular Devices, CA, USA). Alternatively, cells were labeled by anti-Ki-67 antibodies and cycling cells were visualized by immunofluorescence.

For Apoptosis assays, cells were cultured in DMEM medium supplemented with 10% FBS for 24 hours. Cells were treated with or without UV irradiation at 25 J/m^2^, and cultured in serum-free medium. Cells were harvested at the designated time points, stained by propidium iodide prior to flow cytometric analysis of sub-G1 apoptotic fractions (FACSCalibur, CA, USA). Alternatively, apoptosis in the primary tumors were measured by immunostaining of the apoptotic cells using the dUTP-fluorescein TUNEL kit (Roche).

For colony formation assay, 150 cells were seeded into each well of 6-well plates, and the medium was replaced every other day. After twelve days, cells were stained with crystal violet (0.1% in 20% ethanol). The colonies, defined as >50 cells/colony, were counted by ImageJ (National Institutes of Health, MD, USA).

### *In vivo* rat C6 glioma model

Fifty-one 9-week-old male Sprague-Dawley rats (National Laboratory Animal Center of Taiwan) were used. The rats were arbitrarily allocated to receive Ctrl-KD or OPN-KD C6 cells, and assigned to the MRI experiments (n = 3 per group; experiment repeated three times) and survival experiment (n = 6 per group; experiment repeated twice). In addition, nine normal rats (n = 3 each time; experiment repeated three times) were used as controls of proton magnetic resonance spectroscopy (1H MRS). All experimental protocols were approved by the Institutional Animal Care and Utilization Committee (IACUC) at Academia Sinica, Taipei, Taiwan. All experimental procedures were carried out in accordance with approved guidelines of the IACUC. A total of 0.9 × 10^5^ C6 cells (in 0.6 μl PBS) was injected into striatum [Bregma = 0.2 mm, lateral = 3.0 mm, and depth = 5.0 mm] using a 30-gauge needle (Hamilton, NV, USA) and a micro-infusion pump (Model 310; KD Scientific, MA, USA) as described previously^49^.

### Multi-parametric magnetic resonance imaging (MRI) methods

The animals were subjected to three MRI examinations on days 12, 17, and 21 days after implantation, using a horizontal 7.0-T spectrometer (PharmaScan 70/16, Bruker, Germany) with an active shielding gradient of 300 mT/m in 80 μs. Images were acquired using a 72-mm birdcage transmitter coil and a separate quadratic surface coil for signal detection. Each rat was initially anesthetized with 5% isoflurane flowing in O_2_ at 2 l/min. Once the animal was fully anesthetized, the isoflurane was maintained at 1.5 ~ 2.0% to minimize anesthesia-induced hemodynamic fluctuations. The rat was allowed to breathe spontaneously throughout the experiment. The breathing rate was maintained at between 60 and 70 breaths/min. T2WIs were acquired using a fast spin-echo sequence with field of view (FOV) = 2.56 cm, slice thickness = 1 mm, 8 slices, repetition time (TR) = 3000 msec, echo time (TE) = 70 msec, echo train length = 8, number of excitations (NEX) = 12, and matrix size = 256 × 128 (zero filled to 256 × 256). Diffusion-weighted images (DWIs) were obtained with the Stejskal-Tanner spin-echo sequence (TR = 2600 ms, TE = 35 ms, NEX = 1, diffusion gradient duration = 5 ms, diffusion gradient separation = 12 ms, four b values = 0, and 1100 mm^2^/s applied along the X, Y, and Z directions) to generate an averaged apparent diffusion coefficient (ADC) maps by customized MATLAB codes. At the last time point, dynamic contrast-enhanced MRI (DCE MRI) and proton MR Spectroscopy (1H-MRS) were also performed. T2WIs, ADC maps, and DCE MRI were acquired in the same location. The DCE MRI was using a dynamic series of 80 T1-weighted gradient-echo images with TR = 130.2 ms, TE = 4.1 ms, filp angle = 30°, 8 slices, 2D acquisition, FOV = 2.56 cm × 2.56 cm, matrix size = 256 × 128 (zero filled to 256 × 256), slice thickness = 1 mm, and NEX = 1. An intravenous bolus injection of 0.2 mmol/kg Gd-DTPA (Gadoevist, AG, Germany) was administered during acquisition of the eighth image in total 80 images. Single-voxel 1H-MRS is acquired by the point resolved spectroscopy sequence with voxel size = 2.5 × 2.5 × 2.5 mm, TR = 3000 ms, TE = 136 ms, and NEX = 256. For each rat, one volume of interest was localized, centered on the tumor. The carrier frequency for the acquisition of proton MRS in this study was located at the water peak (4.7 ppm).

The contour of the tumors was delineated based on the contrast provided by the T2WIs between tumor and brain tissues. Regions of interest were evaluated by the operator and two experienced observers who were blinded to the treatment groups in order to determine the inter-observer variation. Tumor volume was calculated by summing the area in three dimensions using Avizo software (version 6.0, Visualization Sciences Group, MA, USA). In DCE-MRI, the kinetic analysis of dynamic signal enhancement by Gd-DTPA was based on the compartment model of Tofts^50^. The rate of contrast agent uptake (dC_t_(t)/d*t*) can be given as dC_t_(t)/d*t* = K^trans^ (C_p_–C_t_/V_e_), where K trans is vascular permeability, C_p_ is the concentration of contrast agent in the plasma space, C_t_ is the concentration of contrast agent in the tissue extravascular and extracellular space, and V_e_ is the leakage space per unit volume of measured tissue. Among these parameters, the K^trans^ is sensitive to the regions with higher vascular permeability, which is closely associated with the increased degree of angiogenesis, as demonstrated in our previous study^51^. Optimal values of the pharmacokinetic parameters K^trans^ for each pixel were calculated by fitting the dynamic curve of the tissue signal using nonlinear regression analysis. The general initial T1 value was applied according to our previous study^51^. The arterial input function was not acquired during scanning since it was not required in the calculation. In 1H-MRS, the ratios of metabolite quantifications were performed automatically by LCModel software (Oakville, Canada)^52^. The magnitude calculation was performed on all spectra. The MR spectroscopic data were all displayed in the magnitude mode.

### Histological examination

After the last scan, animals were sacrificed immediately and the brains were removed and embedded with paraffin. Five micron thick brain sections were subjected to hematoxylin and eosin (H&E) staining and immunohistochemistry of OPN, Ki-67, DAPI, RECA, VEGFR2, and VEGF as described^53^. Four rats in each of the OPN-KD and Ctrl-KD groups were selected for quantification. Four representative images (at 40× magnification) were selected from each tumor, and the coverage areas and cell numbers were analyzed by software ImageJ.

### Statistical Analysis

Results obtained from independent experiments were presented as the mean ± standard error of the mean (SEM). Between-group differences were tested with analysis of variance (ANOVA) followed by Fisher’s post-hoc tests. Student’s two-tailed *t*-test was used to determine the difference of means between any two groups. All statistical analyses were performed using StatView software (SAS Institute, NC, USA). A P value of <0.05 was taken as statistically significant.

## Additional Information

**How to cite this article**: Yao, N.-W. *et al*. Functional assessment of glioma pathogenesis by *in vivo* multi-parametric magnetic resonance imaging and *in vitro* analyses. *Sci. Rep*. **6**, 26050; doi: 10.1038/srep26050 (2016).

## Supplementary Material

Supplementary Information
