# Supplementary material for: Functional assessment of glioma pathogenesis by in vivo multi-parametric magnetic resonance imaging and in vitro analyses
Source: Sci Rep. 2016 May 20;6:26050. doi: 10.1038/srep26050 (PMC4873752; doi:10.1038/srep26050)
Supplement: Supplementary Information [file srep26050-s1.pdf]

Functional assessment of glioma pathogenesis by *in vivo* multi-parametric magnetic resonance imaging and *in vitro* analyses

Nai-Wei Yao[1,2], Chen Chang[2], Hsiu-Ting Lin[2], Chen-Tung Yen[1*], and Jeou-Yuan Chen[2,3*]

[1]Department of Life Science, National Taiwan University, Taipei, Taiwan.

[2]Institute of Biomedical Sciences, Academic Sinica, Taipei, Taiwan.

[3]Institute of Genome Sciences, National Yang-Ming University, Taipei, Taiwan.

*Correspondence to

Jeou-Yuan Chen bmchen@ibms.sinica.edu.tw; Chen-Tung Yen ctyen@ntu.edu.tw

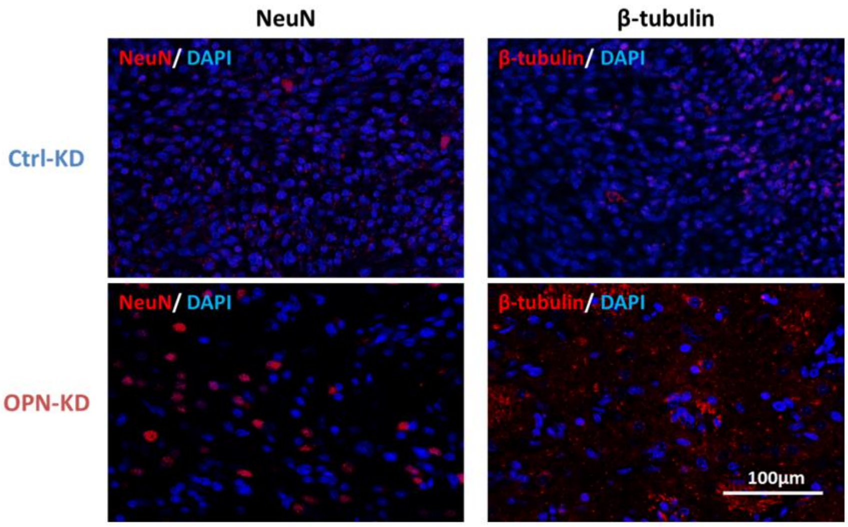

Figure S1. Neuronal markers expression in C6/Ctrl-KD cell- and C6/OPN-KD cell-derived tumors were examined by staining of NeuN and β-tubulin (red) followed by counterstaining with DAPI (blue).

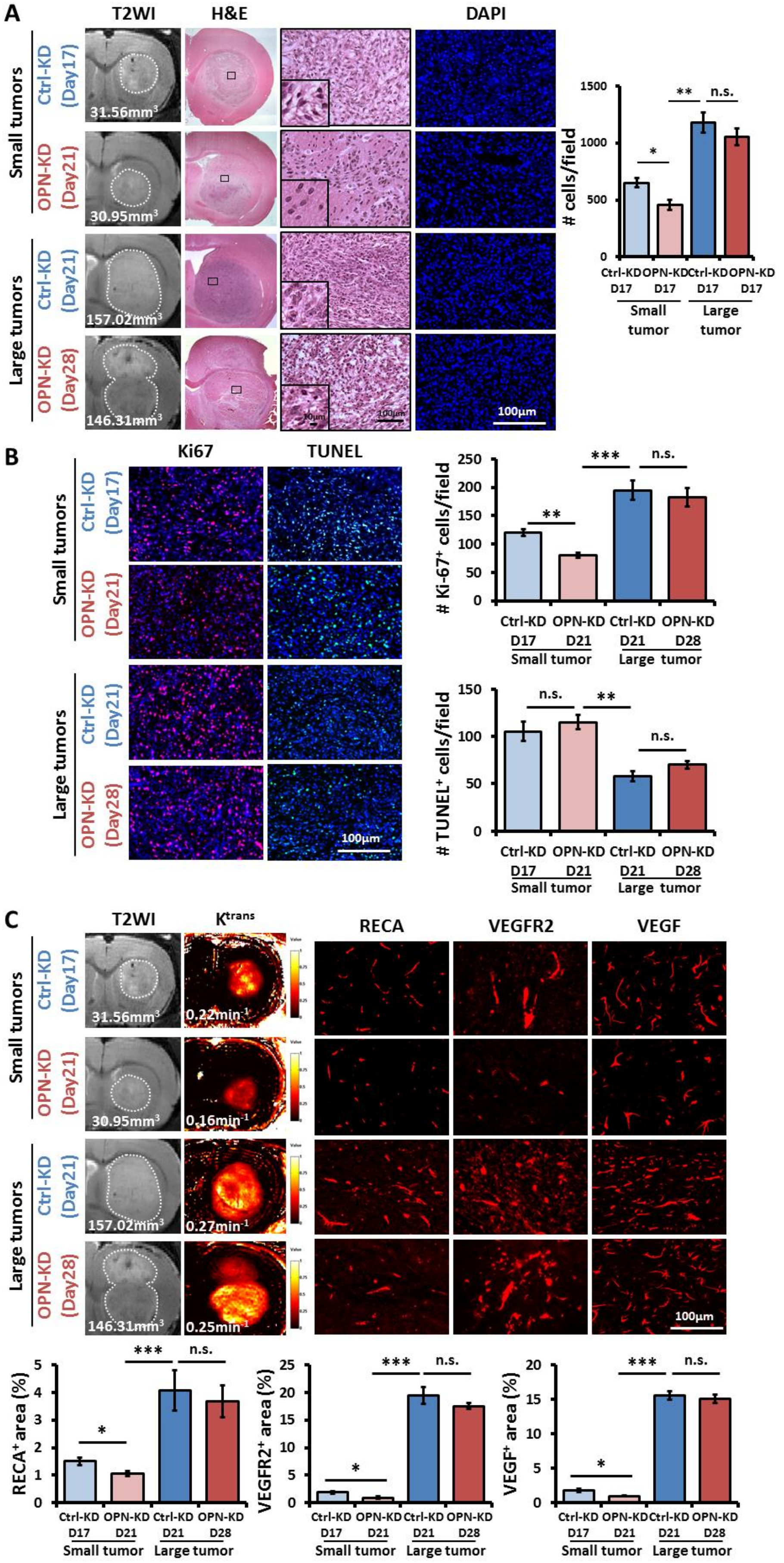
A
T2WI
H&E
DAPI
Small tumors
Large tumors
Ctrl-KD (Day17)
OPN-KD (Day21)
Ctrl-KD (Day21)
OPN-KD (Day28)
31.56mm³
30.95mm³
157.02mm³
146.31mm³
100μm
cells/field
1500
1000
500
0
*
**
n.s.
Ctrl-KD D17
OPN-KD D17
Ctrl-KD D17
OPN-KD D17
Small tumor
Large tumor
B
Ki67
TUNEL
100μm
Ki-67⁺ cells/field
250
200
150
100
50
0
**
***
n.s.
Ctrl-KD D17
OPN-KD D21
Ctrl-KD D21
OPN-KD D28
Small tumor
Large tumor
TUNEL⁺ cells/field
150
100
50
0
n.s.
**
n.s.
C
T2WI
Ktrans
RECA
VEGFR2
VEGF
0.22min⁻¹
0.16min⁻¹
0.27min⁻¹
0.25min⁻¹
100μm
RECA⁺ area (%)
VEGFR2⁺ area (%)
VEGF⁺ area (%)
*
***
n.s.
Ctrl-KD D17
OPN-KD D21
Ctrl-KD D21
OPN-KD D28
Small tumor
Large tumor

Figure S2. KD of OPN retards tumor growth by examining the images and histology-related parameters in C6/Ctrl-KD cell- and C6/OPN-KD cell-derived tumors of similar size at both early and late stages. (A) Representative images of T2WI and H&E staining of C6/OPN-KD- and C6/Ctrl-KD-derived tumors are shown. The nuclear morphology was shown in magnified images in the right panels and in the insets. Tumor cellularity was evaluated by nuclear staining by DAPI. (B) Tumor cell proliferation was examined by staining of Ki-67 (red) followed by counterstaining with DAPI (blue). Tumor cell apoptosis was examined by TUNEL staining (green) followed by counterstaining with DAPI (blue). (C) Representative T2WI and K^{trans} maps of C6/OPN-KD- and C6/Ctrl-KD-derived tumors are shown. The color ranged from black (0/min), orange (0.5/min), to white (1/min). Tumor vessel density was evaluated by staining RECA. Expression of angiogenic markers was examined by staining VEGFR2 and VEGF. All the representative images are shown at 40x magnification. Data are presented as means ± SEM in the bar graph. *, $p<0.05$; **, $p<0.01$; ***, $p<0.001$ by student's two-tailed *t*-test.
